# 

**Published:** 1886-01

**Authors:** 


					﻿1886.
OW<SERIES
Vol. XXV
4* 1855 O'
NEW SERIES.
*■ vol. ii.-ri^.ihBF

EDIGAL^WJ®®
UURNAfe

-C, ri*A>SJptr>'-&t eyerie	i
Zp by* W.F.WESTMORELAND. I®
kA H.V.M.MILLZR.M.D.LL.D.ffl'
pA	JAMES A.GRAY. M.D.
^MUSHED By JAMES P.HARRISON&C®
ut	Atlanta,ga. aeb
SUBSCRIPTION: $2.50 A YEAR.
				

